# Rapid Synthesis of the Literature on the Evolution of Gamification in Distal Radial Fracture Rehabilitation

**DOI:** 10.7759/cureus.29382

**Published:** 2022-09-20

**Authors:** Waqar M Naqvi, Moh'd Irshad Qureshi

**Affiliations:** 1 Physiotherapy, Ravi Nair Physiotherapy College, Datta Meghe Institute of Medical Sciences, Wardha, IND; 2 Neuro-Physiotherapy, Ravi Nair Physiotherapy College, Datta Meghe Institute of Medical Sciences, Wardha, IND

**Keywords:** augmented reality (ar), virtual reality, hand functions, rehabilitation, physiotherapy, gamification, distal radial fractures

## Abstract

Distal radial fractures (DRF) are often encountered in upper limb fractures globally, and their associated complications affect the functional independence of the individual following the injury. The potential of gamification in applied rehabilitation is expanding its horizons in the rehabilitation of conditions ranging from neuromotor deficits to cognitive impairments. However, the synthesis of the literature is aimed at analyzing and summarizing the evolution of gamification in DRF rehabilitation. A comprehensive search and extraction of relevant literature were conducted and reviewed for the applicability of population analysis, interventional methodology, comparative factors, outcome measures, and the type of study. Thirteen studies were included and evaluated, including randomized controlled trials (RCTs), literature reviews, systematic reviews, meta-analyses, and bibliometric analyses. The conclusions demonstrated an improvement with gamification and addressed it as an effective rehabilitation method. Based on the analysis of the data that was extracted, the conclusion supports the use of gamification in the rehabilitation of DRF and looks into how it can help improve the person's functional capacity.

## Introduction and background

A distal radial fracture (DRF) is the most common upper limb fracture that limits the functional activities of daily living (ADLs) and requires precise attention during rehabilitation for an effective return to functional independence [1]. Patients were immobilized for six weeks following closed reduction internal fixation with a K-wire and plaster cast, resulting in disuse atrophy of the forearm and wrist musculature, which ultimately limit functional hand mobility [2]. Conventionally, physiotherapy rehabilitation is advised for post-cast removal to effectively improve motor impairments, including pain, reduced range of motion (ROM), muscle strength, and functional capacity. Conventional therapy is usually painful, tedious, and repulsive, and it takes time for patients to appreciate improvements in maintaining these improvements due to the recoiling property of the elastic muscle fibers [3]. Gamification provides exergaming sessions that are painless, interesting, interactive, engaging, and easy to understand and execute [4]. Recent literature has illustrated the positive impact of the integration of gamification into clinical practice in patients with chronic low back pain [5], lower limb amputation [6], cerebral palsy [7], and traumatic brain injuries [8]; however, its efficacy in post-traumatic fracture rehabilitation is yet to be explored. Hence, gamification is a viable new therapeutic technique that has a wide range of potential applications.

Virtual reality (VR), augmented reality (AR), and mixed reality (MR) provide mature access to rehabilitation technology and are versatile fields for evidence-based clinical practice [9]. The Oculus Quest (metaverse) (Reality Labs, Washington, DC) is a cost-effective, portable, easy-to-access, and easy-to-administer variant of VR technology that may facilitate its widespread adoption in rehabilitation. Gamification induced with Oculus Quest appears to be an easy, non-invasive, painless, engaging, immersive, interesting, and interactive approach that considers flow, meaningful rewards, and finding innovative ways for patient education, assessment, and rehabilitation. It also influences user behavior and motivation through game reminiscence [10]. The analysis of the literature would lay access to explore the evolution of applying gamification to rehabilitation and improving cognitive training skills.

## Review

Research methodology

A combination of words was used for searching the literature in all databases, which included "distal radius fracture" AND "physiotherapy" AND "rehabilitation" AND "virtual reality" AND "gamification." The articles published since 2013 were included in the rapid synthesis of the literature. Articles were searched from PubMed, Emerald, ScienceDirect, SAGE Publications, Wiley, and Google Scholar. The inclusion criteria included the peer-reviewed literature having immersive VR or gamification as an intervention in rehabilitation, a rehabilitative approach to DRF, randomized controlled trials (RCTs), systematic reviews and meta-analyses, and articles published since 2013. The exclusion criteria included non-peer-reviewed literature, non-immersive VR as an intervention, gamification used as a diagnostic method, and articles published prior to 2013. The studies exploring the effectiveness of gamification as an intervention in the rehabilitation of different conditions were given preference.

**Figure 1:**
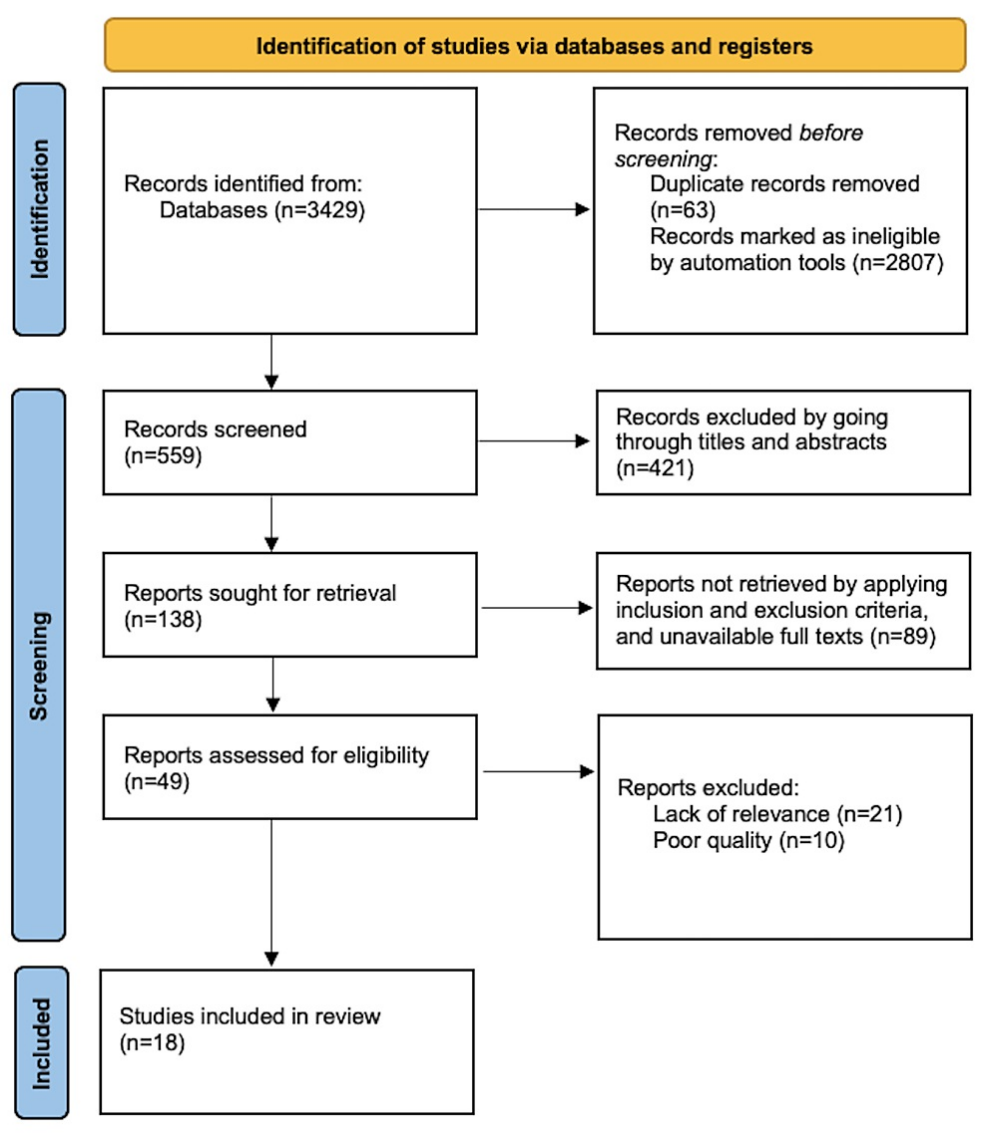
Preferred Reporting Items for Systematic Reviews and Meta-Analyses for rapid review (PRISMA-RR) flow diagram used for searches of databases and data extraction

The literature was extracted from relevant studies and reviewed for the applicability of population analysis, interventional methodology, comparative factors, outcome measures, and the type of study as shown in Table 1. The last search date was considered during extraction to include systematic reviews and meta-analyses. Data extraction was performed by the principal investigator (WMN) and was critically checked by a second reviewer (MIQ). Disagreements were settled through discussion and agreement. Figure 1 shows the Preferred Reporting Items for Systematic Reviews and Meta-Analyses for rapid review (PRISMA-RR) flow diagram used for searches of databases and data extraction.

**Table 1:** Characteristic findings of the research extracted and included in the review

Author	Study title	Study type	Method	Conclusion
MacIntyre and Dewan (2016) [11]	Epidemiology of distal radius fractures and factors predicting risk and prognosis	Review	Literature synthesis	Distal radial fracture is the most common fracture worldwide with a need for rehabilitation to return to functional activities of daily living
Bruder et al. (2013) [12]	Physiotherapy intervention practice patterns used in rehabilitation after distal radial fracture	Observational study	160 distal radial fracture consultation by 14 physiotherapists	Exercise with guidance proves beneficial in recovery after distal radial fracture according to the patient variables and need for functional recovery
Szekeres et al. (2017) [13]	The Effect of Therapeutic Whirlpool and Hot Packs on Hand Volume During Rehabilitation After Distal Radius Fracture: A Blinded Randomized Controlled Trial	Blinded randomized controlled trial	60 patients with clinically healed distal radial fractures divided in two groups received rehabilitation with therapeutic whirlpool and hot packs, respectively	Conventionally hot packs and whirlpool effectively improve the volume of the hands in distal radial fracture patients following functional recovery
Liu et al. (2022) [14]	Virtual Reality Aided Therapy towards Health 4.0: A Two-Decade Bibliometric Analysis	Bibliometric analysis	Mixed research method	Virtual-aided therapy has an impact on the qualitative insights of application and is cost-effective and exploratory in nature, applying gaming elements in healthcare
Yeung et al. (2021) [9]	Virtual and Augmented Reality Applications in Medicine: Analysis of the Scientific Literature	Bibliometric analysis	Bibliometric analysis of 8,399 scientifically proven studies	The clinical practice could be more accurate and precise with the application of virtual reality and augmented reality
Williams and Ayres (2020) [15]	Can Active Video Games Improve Physical Activity in Adolescents? A Review of RCT	Review of randomized controlled trials	Randomized controlled trials and studies within the last 12 years	Exergaming can be a successful tool to develop physical activity in adolescents that could be more adequate and maintainable than numerous established procedures because of its potential and interests
Cugelman (2013) [4]	Gamification: What It Is and Why It Matters to Digital Health Behavior Change Developers	Review	Review on gamification and health behavior change	Gamification seems to impart components in an alike manner to demonstrate health conduct approaches
Brea-Gómez et al. (2021) [5]	Virtual Reality in the Treatment of Adults with Chronic Low Back Pain: A Systematic Review and Meta-Analysis of Randomized Clinical Trials	Systematic review and meta-analysis of randomized clinical trials	14 studies were included in the systematic review and 11 in the meta-analysis	Virtual reality-based rehabilitation can be incorporated into clinical platforms with reference to its impact on the intensity of pain and kinesiophobia
Gumaa et al. (2021) [16]	Validity and Reliability of Interactive Virtual Reality in Assessing the Musculoskeletal System: A Systematic Review	Systematic review	9 studies were included in the quality assessment	Extremely promising validation that interactive virtual reality can be used in persistent neck pain and distal radius fracture
Demers et al. (2021) [7]	Integration of Motor Learning Principles into Virtual Reality Interventions for Individuals with Cerebral Palsy: Systematic Review	Systematic review	26 studies were analyzed and included in the review	Virtual reality-based rehabilitation involves motor learning, putting forth serious gaming as a rehabilitative platform to improve the functional capacity of the upper limbs
Maggio et al. (2019) [17]	Virtual Reality and Cognitive Rehabilitation in People with Stroke: An Overview	Review	Studies performed between 2010 and 2017 that fulfilled inclusion criteria were selected	Virtual reality boosts participation and motivation in rehabilitation by improving cognitive skills involving attention, memory, speech, visual-spatial capabilities, sensory functions, and movement execution
Maggio et al. (2019) [18]	Virtual reality in multiple sclerosis rehabilitation: A review on cognitive and motor outcomes	Review	Studies performed between 2010 and 2017 that fulfilled inclusion criteria were selected	Virtual reality boosts participation and motivation in rehabilitation by improving cognitive skills involving attention, memory, speech, visual-spatial capabilities, sensory functions, and movement execution
Erhardsson et al. (2020) [19]	Commercial head-mounted display virtual reality for upper extremity rehabilitation in chronic stroke: a single-case design study	Single-case design study	7 participants with stroke were included	A combination of commercial gaming, haptic hand controls, and head-mounted virtual reality has an evident effect on the rehabilitation of upper limbs in chronic stroke patients
Huang et al. (2019) [20]	Evaluating the effect and mechanism of upper limb motor function recovery induced by immersive virtual-reality-based rehabilitation for subacute stroke subjects: study protocol for a randomized controlled trial	Study protocol for a randomized controlled trial	60 subacute patients with subacute stroke were assigned in a single-blinded randomized controlled trial	Gamification influences the behavior and motivation of the users using game-reminiscent experiences
Pazzaglia et al. (2020) [21]	Comparison of virtual reality rehabilitation and conventional rehabilitation in Parkinson's disease: a randomised controlled trial	Randomized controlled trial	A single-blinded prospective randomized controlled trial was conducted on 51 Parkinson's patients	An improvement in upper limb function, gait adaptation, complex ambulation skills, and quality of life
Matamala-Gomez et al. (2022) [22]	Impact of virtual embodiment and exercises on functional ability and range of motion in orthopedic rehabilitation	Randomized controlled trial	Three-group controlled trial with 54 patients: 20 patients were in the experimental training group (immersive virtual reality), 20 in the conventional digit mobilization training control group, and 14 in a non-immersive (non-immersive virtual reality) training control group	The immersive virtual reality was exposed to embodied virtual arm for exercising in a rehabilitation program concluding an accelerated functional motor recovery of the fractured arm
Then et al. (2020) [23]	Gamification in rehabilitation of metacarpal fracture using cost-effective end-user device: A randomized controlled trial	Randomized controlled trial	Two-group randomized controlled trial involving 19 patients	Gamification is a safe and cost-effective alternative to conventional programs of rehabilitation
Warsinsky et al. (2021) [24]	Conceptual Ambiguity Surrounding Gamification and Serious Games in Health Care: Literature Review and Development of Game-Based Intervention Reporting Guidelines (GAMING)	Literature review	The two-step research approach in which the first was conducted on a systematic literature review of 206 studies	The outcomes highlighted that explicit definition is lacking. It is heterogenous in nature, followed by the ambiguity of different perspectives and concepts

A study on the epidemiology of DRF predicted risk factors and prognosis and found the responsive factors of chronic disability and pain, which lead to an imperative perspective for therapists to implement more extensive assessment, interventional strategies, and customized educational programs. This leads to the conclusion that DRF is the most common fracture worldwide with a need for rehabilitation to return to functional ADLs [11]. The physiotherapy practice designs exercise and guidance utilized in recovery after DRF patients according to the patient variables and need for functional recovery. This showed conventionally that exercise intervention for patients post-DRF is advised [12]. In a blinded randomized controlled trial (RCT), whirlpool was considered a reliable method of applying heat over the affected site post-DRF. This mentioned that conventionally, hot packs and whirlpool effectively improve the volume of the hands in DRF patients following functional recovery [13].

A bibliometric analysis analyzed two-decade data on VR-aided therapy for health. A blended examination strategy was embraced for this exploration, utilizing a bibliometric investigation where a quantitative technique led to an overview outline of VR-assisted treatment, along with a literature review putting an impact on the qualitative insights of the application. The data cost is added to design further healthcare administrations and associations between healthcare counterparts utilizing elements of the game and presenting new exploration programs [14]. Again, a bibliometric analysis of 8,399 scientifically proven studies on the application of AR and VR supported the emerging versatility of AR and VR in medicine. The in-depth analysis and application of accessibility of technology made a conclusion that clinical practice could be more accurate and precise. VR and AR provide mature access to technology in medicine providing a more versatile field for evidence-based clinical practice [9].

A review of RCTs concentrating on physical activity (PA) improvement in adolescents found that exergaming can be a successful tool to further develop PA in adolescents that could be more adequate and maintainable than numerous established procedures following its potential and interests. The resilience of this review lies in its incorporation of RCTs making it a higher level of evidence. In any case, involving exergaming to advance and impact adherence to PA or elements that obstruct or urge adolescents to play active video games has not yet been determined [15]. Gamification and its importance to digital health behavior have explored the connection between gamification and conducting systems along with models that can be utilized to analyze gamification as a possible facilitating structure to digital interventions. Gamification seems to impart components in an alike manner to demonstrate health conduct approaches signifying rewards, user-friendliness, and exceeding creative ways of making digital interventions engaging [4].

Systematic review and meta-analysis of randomized clinical trials of VR-based treatments for persistent low backache indicated that VR-based rehabilitation could significantly reduce pain intensity and kinesiophobia in chronic low backache patients. However, the application of VR-based rehabilitation is highly heterogeneous and can affect the outcomes. VR-based rehabilitation can be incorporated into clinical platforms with reference to its impact on the intensity of pain and kinesiophobia following short- and mid-term follow-ups in chronic low backache patients [5]. A systematic review analyzed the reliability and validity of interactive VR for the evaluation of the musculoskeletal system. There is extremely promising validation that interactive VR utilizing real-time feedback or games is exceptionally substantial and dependable in analyzing ROM in asymptomatic individuals and patients with persistent neck pain and DRF. The validity of VR should be investigated by evaluating postural balance, response time, movement velocity, and precision to make a powerful determination [16].

The implication of the principle of motor learning in VR-based rehabilitation of patients with cerebral palsy was analyzed in a systematic review of 26 studies, and it was concluded that tailored VR-based rehabilitation programs customized for individual needs and requirements lead to a better functionally independent life. VR-based rehabilitation involves motor learning, including serious gaming as a rehabilitative platform to improve the functional capacity of the upper limbs [7]. It boosts participation and motivation in the rehabilitation of patients with stroke [17] and multiple sclerosis [18] by improving cognitive skills involving attention, memory, speech, visual-spatial capabilities, sensory functions, and movement execution. A single-case design on the application of head-mounted VR-based rehabilitation in a chronic stroke patient reported that a combination of commercial gaming, haptic hand controls, and head-mounted VR has an evident effect on the rehabilitation of upper limbs in chronic stroke patients. The cost-effective values accompanied by wide production made VR-based technologies easily available and accessible for different variants of gamification in rehabilitation [19]. The recovery of motor function of upper limbs following the application of an immersive VR-based rehabilitation program on 60 subacute patients with subacute stroke showed that gamification influences the behavior and motivation of the users using game-reminiscent experiences. Guidelines to use gamification for upper limb rehabilitation have also been provided stating motivation improves compliance [20].

A single-blinded prospective RCT was conducted on 51 Parkinson's patients to compare the efficacy of VR-based rehabilitation programs concluding with an improvement in upper limb function, gait adaptation, complex ambulation skills, and quality of life [21]. The utility of indulging immersive VR in neurorehabilitation by using visual-spatial feedback is supported in different conditions; however, its efficacy in improving functional motor ability was assessed in patients managed conservatively following DRF. The immersive VR was exposed to embodied virtual arms for exercising in a rehabilitation program concluding an accelerated functional motor recovery of the fractured arm [22]. A two-group RCT analyzed improvement in hand function on the implication of gamification with a mobile device tool in metacarpal fracture rehabilitation and concluded it as a safe and cost-effective alternative for a conventional program of rehabilitation [23].

An analysis of ambiguity applied as concepts for serious games and gamification in healthcare reviewed the literature systematically and also developed a guideline for gaming, i.e., Game-Based Intervention Reporting Guidelines. It involved an explicit analysis of 206 studies leading to a result of the application of gaming concepts as serious gaming in healthcare elevation. The outcomes highlighted that explicit definition is lacking more than half of the concept application of serious gaming. It is heterogenous in nature, followed by the ambiguity of different perspectives and concepts [24].

## Conclusions

The analysis of the literature builds an evolutionary process in the development of gamification in rehabilitation. The impact of the integration of gamification has an imperative effect on the physical activity of adolescents, as well as in patients with chronic low back pain, lower limb amputation, cerebral palsy, and traumatic brain injuries along with clinical practice. The expanded horizon of the application of gamification in rehabilitation is expanding on the basis of its efficacy in improving fine and gross motor skills. The immersive environment providing real-time feedback proves promising in improving the compliance and adaptability of long-term patient care. An effective response time, movement velocity, and precision of movement develop using gamification creating a powerful determination in a diseased condition. Integrating the heterogenous, cost-effective, and creative ways of the pertinence, the ambiguity of the application of gamification can be expanded and explored in a wide range, explaining its evolution in different variants in rehabilitation.
